# Association of TDP-43 proteinopathy, cerebral amyloid angiopathy, and Lewy bodies with cognitive impairment in individuals with or without Alzheimer’s disease neuropathology

**DOI:** 10.1038/s41598-020-71305-2

**Published:** 2020-09-03

**Authors:** David X. Thomas, Sumali Bajaj, Kevin McRae-McKee, Christoforos Hadjichrysanthou, Roy M. Anderson, John Collinge

**Affiliations:** 1 MRC Prion Unit at UCL, UCL Institute of Prion Diseases, London, W1W 7FF UK; 2 Department of Infectious Disease Epidemiology, School of Public Health, Faculty of Medicine, Imperial College London, London, W2 1PG UK

**Keywords:** Neurodegenerative diseases, Alzheimer's disease, Statistics, Neuroscience, Neurology

## Abstract

Alzheimer’s disease patients typically present with multiple co-morbid neuropathologies at autopsy, but the impact of these pathologies on cognitive impairment during life is poorly understood. In this study, we developed cognitive trajectories for patients with common co-pathologies in the presence and absence of Alzheimer’s disease neuropathology. Cognitive trajectories were modelled in a Bayesian hierarchical regression framework to estimate the effects of each neuropathology on cognitive decline as assessed by the mini-mental state examination and the clinical dementia rating scale sum of boxes scores. We show that both TDP-43 proteinopathy and cerebral amyloid angiopathy associate with cognitive impairment of similar magnitude to that associated with Alzheimer’s disease neuropathology. Within our study population, 63% of individuals given the ‘gold-standard’ neuropathological diagnosis of Alzheimer’s disease in fact possessed either TDP-43 proteinopathy or cerebral amyloid angiopathy of sufficient severity to independently explain the majority of their cognitive impairment. This suggests that many individuals diagnosed with Alzheimer’s disease may actually suffer from a mixed dementia, and therapeutics targeting only Alzheimer’s disease-related processes may have severely limited efficacy in these co-morbid populations.

## Introduction

Alzheimer’s disease is a progressive neurodegenerative disease displaying extreme variability in clinical features and biomarkers, and with multiple genetic and environmental factors involved in its aetiology and progression^1–3^. Despite this, the current ‘gold-standard’ post-mortem diagnosis of Alzheimer’s disease is primarily an inclusive diagnosis based on the presence of two brain lesions—amyloid plaques and neurofibrillary tangles^4^. While this system effectively identifies patients with pathological changes in both amyloid-β and tau, it does not acknowledge the major role of coincident pathologies commonly observed within ‘Alzheimer’s disease’ populations^5^. Indeed, whilst it is becoming widely appreciated that multiple pathologies are the norm amongst dementia patients^6^, consensus criteria are still narrowly focused on identifying correlations between cognitive impairment and in vivo biomarkers of amyloid-β and tau accumulation^7^, with investigations into common co-pathologies given low priority at present. This strategy ignores the likelihood that even within well-defined ‘Alzheimer’s disease’ populations, in which all individuals possess substantial amyloid-β and tau pathology, a large proportion of the observed cognitive decline may still be driven by alternative pathologies due to their high prevalence and potency.

Previous clinico-pathological correlation studies have confirmed that many non-Alzheimer’s neuropathologies are associated with an increased probability of having a clinical dementia diagnosis at death^8,9^, lower final MMSE scores at death^10^, and that these deleterious effects typically increase over time as an individual approaches death^11^. Furthermore, the proportion of cognitive decline attributable to any one neuropathology varies significantly at the individual level^12^. However, no study has yet compared the association of common neuropathologies with pre-mortem cognitive decline in the presence vs absence of concomitant Alzheimer’s disease.

To tackle these concerns, we used longitudinal cognitive scores and post-mortem neuropathological data from the National Alzheimer's Coordinating Center (NACC) database to investigate the pre-mortem cognitive trajectories of patients with and without Alzheimer’s disease neuropathological change (ADNC). In order to robustly examine the most common neuropathologies known to associate with cognitive impairment, we excluded all individuals with rare neuropathologies which may associate with substantial cognitive impairment but were too low in number to statistically examine, and those with rare mutations in any gene related to dementia. We then used Bayesian Hierarchical regression models to estimate the association of Alzheimer’s disease neuropathology, TDP-43 proteinopathy, cerebral amyloid angiopathy (CAA), and Lewy bodies with cognitive trajectories after accounting for the covariates consisting of demographic features, and other neuropathologies. We focused on these three pathologies due to their high prevalence, clearly defined and widely accepted protocols for neuropathological examination, and high evidence in the literature of an association with cognitive impairment.

## Methods

### Data source

Data used in this study were obtained from the National Alzheimer’s Coordinating Center (NACC), which co-ordinates data collection across a network of 39 past and present Alzheimer’s Disease Centers (ADCs) across the USA. ADC participants complete roughly annual clinical evaluations according to a standardized protocol, with data collected in the Uniform Data Set (UDS)^13^, which comprises detailed assessments including physical and neurological exams, a neuropsychological test battery, and questionnaires that assess neuropsychiatric symptoms, family history, functional abilities, medical history, and medication use. A subset of participants who consent to autopsy undergo standardised neuropathological examination which contribute to the Neuropathology Data Set. UDS data collected from September 2005 to December 2018 were used in this study. Research using the NACC database is approved by University of Washington Institutional Review Board and authors who access the data are required to sign and comply with the data use agreement. Written informed consent was obtained from all participants and informants with their anonymity preserved.

### Clinical and cognitive assessments

Cognitive and clinical measures reported here are the mini mental state examination (MMSE) and the Clinical Dementia Rating scale sum of boxes (CDR-SB). Both the MMSE and CDR were administered and scored by trained staff within their respective Alzheimer’s disease centers.

The MMSE is a commonly administered 30-point test of cognitive status across the domains of orientation to time and place, concentration, attention, verbal learning, naming, and visuoconstruction^14^. Despite several weaknesses including poor sensitivity for mild cognitive impairment, it has been utilised extensively in both research settings and in clinical trials.

The Clinical Dementia Rating scale (CDR) is an operationalized measure of cognitive and functional ability derived from interviews by a clinician with the patient and a second informant, and rates impairment across the categories of memory, orientation, judgement and problem solving, community affairs, home and hobbies, and personal care^15^. Each category is rated on severity of impairment on a five-point scale in which none = 0, questionable = 0.5, mild = 1, moderate = 2 and severe = 3. These 5 scores can then be summed to produce a “Sum-of-boxes” score. In this work we utilised the CDR Sum-of-boxes score for analyses.

### Neuropathological methods and measures

Neuropathological examinations were performed using standardised methods at each centre in accordance with relevant guidelines and regulations, with data collected via a standardised neuropathology form and coding guidebook^4,16^. Briefly, gross inspection of brain was used first to assess regional atrophy and cerebrovascular disease, followed by individual sampling of brain regions including the medulla, pons, midbrain, cerebellar cortex, thalamus, basal ganglia, hippocampus, cingulate, amygdala, middle frontal gyrus, superior & middle temporal gyri, inferior parietal lobule, and occipital cortex. All brain regions were stained with hematoxylin and eosin, with select regions given additional stains for specific neuropathologies. Tau was most commonly labelled using PHF1 or AT8. Amyloid-β was most commonly labelled using 4G8, 10D5, or thioflavin-S. Both TDP-43 and α-synuclein were stained using a range phospho-specific and non phospho-specific antibodies which varied by disease center.

While the NACC standardised neuropathology form has been revised multiple times since its inception, only the most recent version (version 10) includes data on the presence of regional TDP-43 pathology. Therefore, the only individuals included in this study were those who had been examined using version 10 of the Neuropathology form, and thus there was no need to harmonise any neuropathological variables between older form versions. The following Neuropathology variables were used in this study: NACCAMY, NACCINF, NPADNC, NPLBOD, NPTDPA, NPTDPB, NPTDPC, NPTDPD, NPTDPE, NPPDXA, NPPDXB, NACCPRIO, NPPDXD, NPPDXE, NPPDXF, NPPDXG, NPPDXH, NPPDXI, NPPDXJ, NPPDXK, NPPDXL, NPPDXM, NPPDXN, NPFTDTAU.

### Sample selection

Between September 2005 and December 2018, 39,412 participants were recruited by clinicians and investigators at 39 Alzheimer’s disease centers across the United States for longitudinal assessment using varying sampling strategies at each center. We requested all data from NACC in the December 2018 data freeze. We then selected the 924 individuals who were followed to autopsy and had complete neuropathological data available for parenchymal amyloid-β pathology, Tau pathology (NPADNC variable in NACC), TDP-43 proteinopathy (scored in the amygdala NPTDPB, hippocampus NPTDPC, entorhinal cortex NPTDPD and neocortex NPTDPE), Lewy bodies (NPLBOD), and CAA (NACCAMY). 223 Individuals were then excluded if they had a positive test for any familial dementia gene, or if neuropathological examination revealed any non-amyloid angiopathy, pigment-spheroid degeneration, multiple system atrophy, prion disease, trinucleotide disease, any malformation of cortical development, a metabolic/storage disorder of any type, leukodystrophy, multiple sclerosis, traumatic brain injury, neoplasm, an infectious process of any type within the brain, herniation at any site, Pick’s disease, corticobasal degeneration, progressive supranuclear palsy, chronic traumatic encephalopathy, amyotrophic lateral sclerosis, or any other atypical tau pathology. We then excluded another 73 individuals who did not have MMSE scores in the range 0–30 and years of education between 0–36 (allowable codes by NACC). And finally we only retained 574 individuals with complete measurements on MMSE, CDR-SB, neuropathological variables mentioned above and the covariates that we control for as mentioned in the statistical analysis (see Supplementary Figure S4 online).

### Operationalisation and binary categorisation of neuropathological variables

Binary categories were created for Alzheimer’s disease neuropathological change, Lewy bodies, CAA, and TDP-43 proteinopathy using cut-off values which led to reasonably balanced groups. For Alzheimer’s disease neuropathological change, individuals were classed as positive (ADNC +) if they satisfied criteria for “intermediate” or “high” Alzheimer’s disease neuropathological change according to the NIA-AA guidelines (NPADNC = 2 or NPADNC = 3), or as negative (ADNC-) if they possessed zero or low levels of Alzheimer’s disease neuropathological change (NPADNC = 0 or NPADNC = 1)^4^. For Lewy bodies, individuals were classed as positive if they showed any evidence of brainstem, limbic, neocortical, or amygdala Lewy bodies according to the Consortium on Dementia with Lewy Bodies criteria (NPLBOD = 1, 2, 3, 4 or 5)^17^. For CAA, individuals were classed as positive if they possessed moderate or severe amyloid-β positivity in parenchymal and/or leptomeningeal vessels (NACCAMY = 2 or NACCAMY = 3). For TDP-43 proteinopathy, individuals were classed as positive if they possessed any form of immunoreactive TDP-43 inclusions in the amygdala (NPTDPB = 1), hippocampus (NPTDPC = 1), entorhinal/inferior temporal cortex (NPTDPD = 1), or neocortex (NPTDPE = 1). For TDP-43 proteinopathy classification, individuals were designated either as frontotemporal lobar dementia (NPFTDTDP = 1) or limbic-predominant age-related TDP-43 encephalopathy (all TDP-43 positive individuals with NPFTDTDP = 0).

### Statistical analyses

We modelled the association between scores collected at more than one time point for a defined patient (longitudinal scores) of cognition (MMSE and CDR-SB) and the binary categories of neuropathologies using linear mixed effect models in a Bayesian framework. The models included random intercepts and random slopes to account for the repeated measurements of individuals at different points in time, allowing each individual’s trajectory over time to deviate from the overall trend. The analysis included age at death (years), binary *APOE* ε4 allele status (where presence of either one or two *APOE* ε4 alleles is defined as positive), sex, education level (years), and the presence of infarcts and lacunes as covariates to control for potential confounding. Time (in years) was coded as time at visit—time at death, such that the intercept in Fig. 2 (time = 0) corresponds with the time at death. To improve the model fit we included a quadratic term to allow for a quadratic trend. To assess the association between a co-pathology and cognitive decline in ADNC + individuals, we added two-way interaction terms between time and each binary co-pathology in turn. Three-way interactions were also conducted to investigate the difference in the association between co-pathologies and cognitive trajectories in ADNC + and ADNC-individuals.

To infer about the proportion of ADNC + people with substantial TDP-43, CAA or both in our population, we report the point estimate along with the corresponding 95% confidence interval (CI).

Our outcomes MMSE and CDR-SB are bounded (0–30 and 0–18 where lower and higher scores correspond to worse cognition respectively) and assuming that the errors follow a normal distribution may not be appropriate. Hence we conducted sensitivity analysis by considering mixed effects beta regression where the random errors follow a beta distribution^18^. This model performed better in visual posterior predictive checks (see Supplementary Figure S3 online) and the results and their interpretation from the beta regression were in agreement with the linear mixed models and hence we report parameter estimates from the latter for ease of interpretation. Details of the analysis, including the use of diffuse priors for our parameters, are provided in the Supplementary Methods online. Data formatting and plotting was performed in R version 3.5.0 and R studio version 1.1.463. The posterior distributions and 95% Bayesian credible intervals (BCI) were estimated using Hamiltonian Monte Carlo (HMC) through the RStan interface 2.17.3^19^ which uses the “No-U-Turn-Sampler”. The Gelman-Rubin statistic Rhat < 1.1 was used as the default requirement for convergence of parameter chains. After controlling for confounding, the posterior mean estimates corresponding to the covariates are reported as β_MMSE_ (95% BCI) and β_CDR-SB_ (95% BCI) for MMSE and CDR-SB models respectively.

### Methods statement

All methods in this manuscript were carried out in accordance with relevant guidelines and regulations.

## Results

Between September 2005 and November 2018**,** 39,412 individuals completed baseline evaluation at 39 NIA Alzheimer’s disease centers across the United States. Among these participants, 5,512 (58%) of 9,391 deceased participants had an autopsy, and 924 (17%) individuals with an autopsy had complete neuropathological data available for Alzheimer’s disease neuropathological change (ADNC), TDP-43 proteinopathy, Lewy bodies, and cerebral amyloid angiopathy (CAA).

After excluding patients with known familial mutations in genes relating to dementia, other cases of rare dementias, and other exclusions based on data availability (see “Methods”), 574 patients were selected for downstream analyses. Participants underwent an average of four assessments for both MMSE and CDR-SB.

Among the 436 ADNC + individuals, 340 (78%) had at least one other neuropathology visible at autopsy, and it was more common to have two or more co-pathologies (157/436, 36%) than to have none (96/436, 22%).

Using the chi-square test for sex, parametric t-test and non-parametric alternative Mann–Whitney U test for age at death and years of education, we found no significant difference between ADNC + and ADNC- groups at 5% level of significance. The number of people with at least one *APOE* ε4 allele was significantly different in the two groups (see Table 1 for p-values and details).

**Table 1:** Demographic features of study participants.

	High/moderate Alzheimer’s disease neuropathological change group (ADNC +)	Low/zero Alzheimer’s disease neuropathological change group (ADNC-)	*P* value^a^
Participants	436 (76%)	138 (24%)	–
Age at death (years)	70.7 (9.3)	70.3 (12.0)	0.74
Sex (female)	194 (44.5%)	57 (41.3%)	0.51
Education (years)	15.6 (3.0)	15.1 (3.5)	0.13
At least one *APOE* ε4 allele	269 (61.7%)	23 (16.7%)	< 0.001
MMSE at last visit	16.6 (7.7)	22.8 (7.3)	< 0.001
CDR-SB at last visit	8.4 (4.9)	4.5 (4.8)	< 0.001
Average number of visits	4.0 (2.3)	4.3 (2.5)	0.22
Average follow-up time (years)	3.4 (2.5)	3.8 (2.8)	0.11
Average time between last visit and death (years)	3.7 (2.3)	2.8 (2.0)	< 0.001

^a^Parameteric chi square and t-test for difference in proportions and means respectively.

*MMSE* mini mental state examination, *CDR-SB* clinical dementia rating scale sum of boxes.

**Figure 1:**
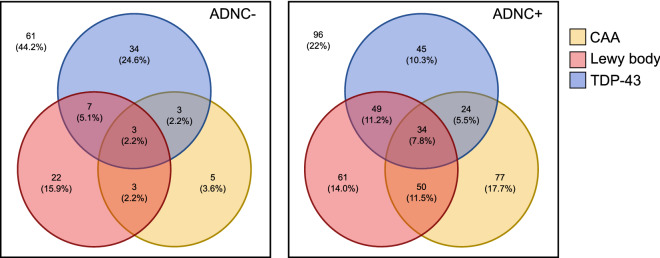
Venn diagram of the prevalence of CAA, Lewy bodies, and TDP-43 proteinopathy in the presence of different severities of Alzheimer's disease neuropathological change. *ADNC* Alzheimer’s disease neuropathological change, *CAA* cerebral amyloid angiopathy. Percentages represent the proportion of ADNC− (left) or ADNC + (right) individuals with the indicated co-pathological signature.

The prevalence of TDP-43 proteinopathy was identical in the ADNC + (152/436, 34%) and ADNC− (47/138, 34%) groups (Fig. 1). CAA was more prevalent in ADNC + individuals (185/436, 42%) than in ADNC− individuals (14/138, 10%).

The most common form of TDP-43 proteinopathy in ADNC- individuals within this population was frontotemporal lobar dementia (31/47, 65%), whereas in ADNC + individuals by far the most common TDP-43 proteinopathy was limbic-predominant age-related TDP-43 encephalopathy (142/151, 94%). In our study dataset we found that almost all individuals diagnosed with frontotemporal lobar dementia had neocortical TDP-43 deposits (37/40, 92%), whereas neocortical TDP-43 proteinopathy was rare in limbic-predominant age-related TDP-43 encephalopathy cases (20/158, 12%).

**Figure 2:**
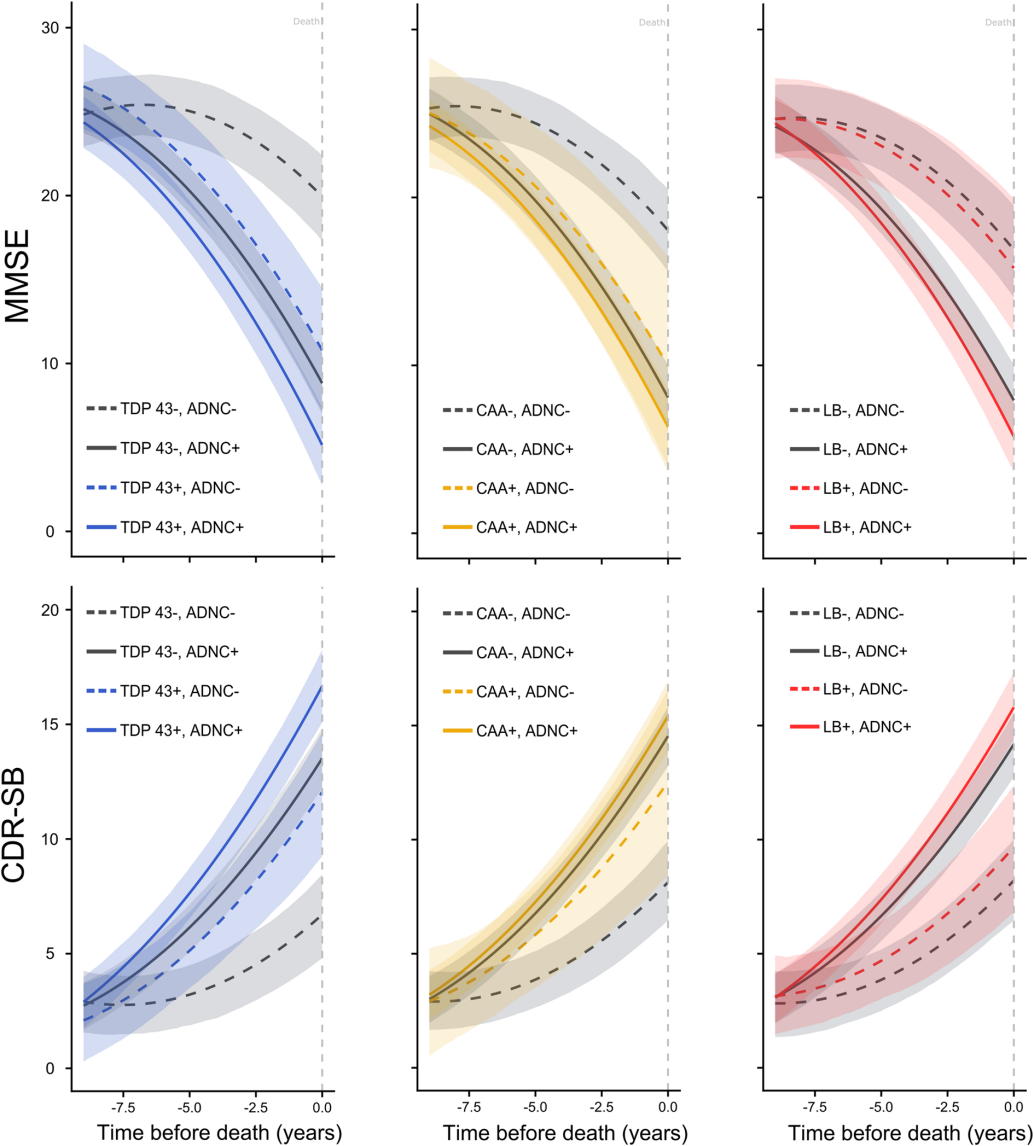
Trajectories of cognitive decline in individuals with or without Alzheimer’s disease neuropathological change and other neuropathological changes at autopsy. Scores have been predicted using a hierarchical model with random intercepts and slopes for the following values of the covariates—72.5 years age at death, APOE ε4 positive, Male, 15.5 years of education, positive for the presence of some infarcts or lacunes, with remaining two co-pathologies positive. Solid and dashed curves are the expected cognitive scores. Shaded regions represent 95% Bayesian credible intervals. *MMSE* mini mental state examination (low score = worse cognition). *CDR-SB* clinical dementia rating scale sum of boxes (high score = worse cognition). *ADNC* Alzheimer’s disease neuropathological change. *CAA* cerebral amyloid angiopathy. *LB* lewy bodies. In the models with three-way interactions our reference category was ADNC− and absence of a specific co-morbid pathology (similar to above, one model each for TDP-43, CAA and Lewy Bodies). We found that in ADNC− individuals, having TDP-43 proteinopathy (β_MMSE_ = − 1.19, 95% BCI (− 1.71, − 0.69); β_CDR-SB_ = 0.70, 95% BCI (0.35, 1.06)) or CAA (β_MMSE_ = -0.89, 95% BCI (− 1.67, − 0.05); β_CDR-SB_ = 0.50, 95% BCI (− 0.02, 1.04)) was associated with a steeper rate of cognitive decline compared to the corresponding reference category, while the presence of Lewy bodies (β_MMSE_ = − 0.12, 95% BCI (− 0.62, 0.40); β_CDR-SB_ = 0.11, 95% BCI (− 0.24, 0.45)) was not associated in the same manner. The association between the presence TDP-43 proteinopathy or CAA with cognitive decline in ADNC− individuals was of similar magnitude to the association between being ADNC + in the absence of TDP-43 proteinopathy (β_MMSE_ = − 1.28, 95% BCI (− 1.59, − 0.96); β_CDR-SB_ = 0.78, 95% BCI (0.56, 0.99)) or CAA (β_MMSE_ = − 1.07, 95% BCI (− 1.40, − 0.75); β_CDR-SB_ = 0.70, 95% BCI (0.48, 0.92)). The estimated proportion of ADNC + individuals that also have substantial TDP-43 proteinopathy, CAA, or both is 0.63, 95% CI (0.59, 0.68). Bayesian beta-regression models were produced as sensitivity analyses corresponding to each previous model. In almost all cases, the directionality and strength of association (measured by the width of 95% BCIs and inclusion of the null value zero) of our results were confirmed in the beta-regression model (see Supplementary Table S1 and Supplementary Table S2 online).

In ADNC + individuals, we assessed if the rate of cognitive decline was the same in the presence or absence of a co-pathology, using one model each for TDP-43, CAA and Lewy Bodies. We estimated the two way interactions of TDP-43 proteinopathy and time (β_MMSE_ = − 0.34, 95% BCI (− 0.64, − 0.04); β_CDR-SB_ = 0.33, 95% BCI (0.13, 1.47)), CAA and time (β_MMSE_ = − 0.04, 95% BCI (− 0.42, 0.35); β_CDR-SB_ = 0.09, 95% BCI (− 0.11, 0.29)) and Lewy bodies and time (β_MMSE_ = − 0.29, 95% BCI (− 0.57, − 0.01); β_CDR-SB_ = 0.19, 95% BCI (− 0.01, 0.39)) (Fig. 2). These results suggest that ADNC + individuals with these pathologies have a steeper rate of cognitive decline compared to those without co-morbid pathologies.

## Discussion

In this retrospective study of individuals subjected to repeated cognitive testing over time (longitudinal study), we found that the presence of TDP-43 proteinopathy or CAA at autopsy each were independently associated with rates of cognitive decline similar to those associated with being ADNC + . Furthermore, the presence of TDP-43 proteinopathy was associated with a more severe worsening of cognitive decline in ADNC- individuals than ADNC + individuals. As 63% (279/436) of ADNC + individuals also had substantial TDP-43 proteinopathy, CAA, or both, it is possible that more than half of individuals given this ‘gold-standard’ diagnosis of Alzheimer’s disease within this population may actually have suffered cognitive decline equally driven by these underappreciated co-pathological mechanisms.

Our results broadly agree with previous studies individually investigating each of these neuropathologies. In individuals with Alzheimer’s disease neuropathology, TDP-43 proteinopathy is associated with more severe cognitive impairment^20^, smaller hippocampal volumes^21^, and increased odds of a patient displaying an amnestic syndrome^22^. TDP-43 proteinopathy also presents in frontotemporal lobar dementia (FTLD) and amyotrophic lateral sclerosis, with a causative role in the latter cemented by the association of various mutations in TDP-43 with familial amyotrophic lateral sclerosis^23^. Recent consensus criteria have been formulated which classify individuals with TDP-43 deposition under the umbrella term ‘limbic-predominant age-related TDP-43 encephalopathy’ (LATE) if they do not fulfil diagnostic criteria for FTLD^24^. While LATE is associated with advanced age and limbic-predominant TDP-43 pathology^24^, there are currently no defined pathological features differentiating between LATE and FTLD individuals. Thus, in our data we defined all individuals with a positive diagnosis of FTLD as FTLD individuals, and all individuals with TDP-43 proteinopathy but no FTLD diagnosis as LATE individuals. Using these definitions, LATE was the most common form of TDP-43 proteinopathy in ADNC + individuals, whereas FTLD was the most common form of TDP-43 proteinopathy in ADNC- individuals. However, the boundary between LATE and FTLD is poorly defined, and there is as yet limited evidence that they are distinct disease processes. Instead, they could either represent different stages of a continuous disease process, or different manifestations of a single disease process occurring in the presence (LATE) or absence (FTLD) of co-morbid Alzheimer’s disease. In any case, processes relating to TDP-43 deposition represent independent pathological mechanisms, which are co-morbid in 19–57% of individuals given the ‘gold-standard’ neuropathological diagnosis of Alzheimer’s disease^25^.

CAA is also associated with increased odds of Alzheimer’s disease dementia^26^, worse episodic memory and perceptual speed scores^27^, and lower overall cognitive ability^28^. Similar to previous reports^26,29^, we found that CAA associated with additional cognitive impairment even after accounting for vascular infarcts and lacunes. While almost ubiquitous within Alzheimer’s disease cases, CAA is not a required feature for diagnosis and its severity varies enormously. CAA therefore represents an additional potent cause of cognitive impairment which is highly variable within the Alzheimer’s disease population.

Similar to previous reports, we found that the additional presence of Lewy bodies associated with worse cognitive impairment in ADNC + individuals^30–33^. However, our data showed no association of Lewy bodies with cognitive impairment over time in ADNC- individuals. This could be due to our low cut-off for positivity of Lewy bodies, as positive identification of Lewy bodies in any brain region was sufficient for Lewy body positive classification, whereas other studies have suggested only widespread neocortical Lewy bodies significantly associate with cognitive impairment^10,34^. However, using neocortical Lewy bodies as the cut-off for Lewy body positivity in our dataset led to very small group sizes, and our primary aim was to robustly investigate the association of highly common coincident pathologies in Alzheimer’s disease. Thus we suggest that while a severe burden of Lewy bodies may have a substantial impact on cognitive impairment, such a severe burden is rare. It should also be highlighted that our comparisons are based on MMSE and CDR-SB tests, which would likely not be sensitive to the psychiatric, extrapyramidal, and hallucinatory symptoms commonly associated with Lewy bodies^35–38^.

Previous studies have also used multi-variable models to assess the independent associations between various neuropathologies with late-life cognitive decline, several of which further support independent associations between TDP-43 proteinopathy and CAA with cognitive decline in cognitively impaired individuals^8–11^.

One common strategy in these studies has been to investigate the association of autopsy-confirmed neuropathologies with either clinical diagnosis of Alzheimer’s disease at the time of death^8,9^ or final cognitive test scores at the time of death^10^. Such models allow for the simultaneous examination of a wide range of neuropathologies in specific brain regions, and has provided strong evidence that isocortical neurofibrillary tangles and lewy bodies are potently associated with low final MMSE scores^10^, and that the combination of vascular, amyloid, tau, neocortical Lewy body, and TAR DNA-binding protein 43 (TDP-43) pathology may account for all of the variance in risk of dementia diagnosis at death that was previously attributed to age^9^. Furthermore, several of these studies have shown that individuals diagnosed with dementia are more likely to have multiple neuropathologies than any one single neuropathology^39^. However, these methods provide only a snapshot of associations at death, and do not provide insight into the association of neuropathologies with the overall trajectory of cognitive decline during the years prior to death. In this work we therefore used mixed linear models which allowed us to investigate the impact of neuropathologies on cognitive scores across time during the decade before death.

Other studies which have examined the relationship between neuropathological signatures and pre-mortem trajectories of cognitive decline have also shown that Alzheimer’s disease pathology, Lewy bodies, and TDP-43 pathology are independently associated with worse pre-mortem trajectories of cognition^11,12,40^. Furthermore, these deleterious associations appear to increase over time^11^, and the proportion of cognitive impairment attributable to each pathology varies widely at the individual level^12^. While these studies provide valuable insights into the independent associations of ADNC and other neuropathologies with pre-mortem cognitive impairment, they do not directly compare the impact of common co-pathologies on pre-mortem cognitive trajectories in the presence vs absence of concomitant ADNC.

Therefore, in this analysis we focused on comparing the associations between TDP-43 proteinopathy, CAA, and Lewy bodies with cognitive impairment over time in individuals with high versus low levels of Alzheimer’s disease neuropathological change. This led to a key finding that 63% of individuals in this population given the ‘gold-standard’ neuropathological diagnosis of Alzheimer’s disease in fact possessed one or more co-pathologies each of which we have directly shown is associated with substantial cognitive decline even in the absence of concomitant Alzheimer’s disease. Under the assumption that these associations are representative of true causal relationships, this observation has several important implications.

First, trial inclusion criteria based solely on cognitive impairment and biomarkers for amyloid-β and tau may still select for a hugely heterogeneous population, even if these biomarkers ensure that 100% of the trial participants go on to develop substantial amyloid-β and tau neuropathology by death. More precisely, over half of such a population may possess additional pathologies which could lead them to continue to decline at a similar rate even if all the disease-associated processes relating to parenchymal amyloid-β and tau pathology were halted, assuming these co-morbid pathologies are independent and/or autonomous once initiated. Excluding individuals that are likely to develop TDP-43 proteinopathy or severe CAA could therefore produce much more homogeneous populations which could allow for smaller and more cost-effective trials. Validating in vivo biomarkers for these co-morbid pathologies should become an urgent priority for the Alzheimer’s disease research community.

Second, it is crucially important for research purposes that post-mortem tissues from neurodegenerative disease cases are recorded with full details of the neuropathologies present in brain at autopsy, and that these details are included in any resultant manuscripts. Many studies have been published which compare brain homogenate from “Alzheimer’s disease” and “Control” patients, defined only by a “neuropathological diagnosis”, or by neuropathological scores for amyloid-β and tau^41–44^. For proper interpretability of the data, we suggest all such studies should also report the levels of TDP-43 proteinopathy, CAA, Lewy bodies and other co-pathologies present in the brain at autopsy. Where such data are not available, this should be clearly stated to make it clear that cases with confounding pathologies have not been excluded.

Whilst these conclusions are striking, there are a number of limitations in this study that require consideration. Our method of dichotomizing each neuropathological score simplifies the complex reality, in which a continuous range of severities are observed for each neuropathology in morphologically and spatially heterogeneous patterns. Further, it likely leads to classification errors for individuals near to the cut-off for positivity, which may be amplified by inevitable variability in classification between different neuropathologists and at different Alzheimer’s disease centers. Neuropathological methods also vary between centres, such as the use of different antibodies and stains for certain proteins, and this may lead to some variability in neuropathological characterisation. Similarly, despite attempts to normalise clinical procedures across Alzheimer’s disease centers, there is still variation in clinical protocols. Further, while our decision to exclude those with rare neuropathologies was necessary for robust examination of our key pathologies of interest, this exclusion means our sample less accurately reflects a clinical sample, and instead reflects a clinical sample in which those with rare neuropathologies have been excluded. We have also assumed that the presence of TDP-43 and Lewy bodies at autopsy signifies a long pre-mortem pathological cascade involving these proteins, which is currently unproven due to the lack of effective in vivo biomarkers for these pathologies. Our data also only represent associations between neuropathological signatures and cognitive decline, and thus we have not proven that true causal relationships exist. Instead, it is possible that such associations are either spurious or epiphenomena, and more research is needed to identify the precise causal pathways that may connect the formation of neuropathological lesions with cognitive impairment. Finally, there is selection bias within our study population, which varies according to the recruitment strategy used by each Alzheimer’s disease centre. The NACC population is also drawn from a population including several dementia clinics, and is therefore most representative of a clinic-based population, and this data is likely to overestimate cases of atypical dementias such as frontotemporal lobar dementia. In addition, our results are only generalizable to a population who consent for autopsy, which were more likely to be caucasians, males, married, and more highly educated in our dataset. Other studies have also reported differences in characteristics of autopsied and non-autopsied individuals^45,46^.

However, it is worth highlighting the results presented here are based on the ‘gold-standard’ neuropathological diagnosis of Alzheimer’s disease, which is by definition more sensitive and specific than any in vivo biomarker of amyloid-β or tau currently available. Thus, even if the true associations between TDP-43 proteinopathy or CAA and cognitive decline are substantially less than we estimate here in neuropathologically confirmed individuals, the implications of these pathologies could be amplified in any clinical trial population selected based solely on cognitive impairment and imperfect in vivo biomarkers. We believe this paper thus provides strong evidence that human studies of dementia need to account for a range of pathological mechanisms, even if their aim is only to treat or study a single pathological process. Such studies would enable investigators to either exclude individuals with confounding pathologies, or investigate the independent impacts of each pathology by stratification at the data analysis stage. To reach this goal, it should become a primary aim within the Alzheimer’s disease research community to aid in the discovery and validation of in vivo biomarkers for co-morbid pathologies commonly present within “Alzheimer’s disease” populations.

## Supplementary information

Supplementary file1

## Data Availability

The corresponding author had full access to all the data in the study and takes responsibility for the integrity of the data and the accuracy of the data analysis. The datasets that support the findings of this study are available from the National Alzheimer’s Coordinating Center at alz.washington.edu on the condition of signing the NACC Data Use Agreement.
