# Supplementary material for: Association of TDP-43 proteinopathy, cerebral amyloid angiopathy, and Lewy bodies with cognitive impairment in individuals with or without Alzheimer’s disease neuropathology
Source: Sci Rep. 2020 Sep 3;10:14579. doi: 10.1038/s41598-020-71305-2 (PMC7471113; doi:10.1038/s41598-020-71305-2)
Supplement: Supplementary file 1 — Supplementary file1 [file 41598_2020_71305_MOESM1_ESM.pdf]

Association of TDP-43 proteinopathy, cerebral amyloid angiopathy, and Lewy bodies with cognitive impairment in individuals with or without Alzheimer's disease neuropathology

David X. Thomas, PhD 1†; Sumali Bajaj, MSc 2†; Kevin McRae-McKee, MSc 2; Christoforos Hadjichrysanthou, PhD 2; Roy M. Anderson, PhD 2; John Collinge, MD 1

Supplementary material

Supplementary Methods

Supplementary Table S1 - Hierarchical linear and beta regression estimates for comparing the effect of neuropathology groups on MMSE over time

Supplementary Table S2 - Hierarchical linear and beta regression estimates for comparing the effect of neuropathology groups on CDR-SB over time

Supplementary Figure S3 - Posterior predictive checks comparing normal vs beta distribution assumptions for MMSE and CDR-SB scores

Supplementary Figure S4 – Sample selection flow chart

Supplementary Methods

For each of the three binary neuropathologies (TDP-43, CAA and Lewy Bodies) we implemented hierarchical linear and beta regression models to understand their association with cognitive decline as measured by MMSE and CDR-SB. Two way interactions with time to death (negatively measured in years) as well as three way interactions with time and binary ADNC pathology were performed. Details of the model and distribution assumptions are as follows (examples for TDP-43):

Two-way interaction with time (Model A)

$$CogScore_{ij} = \beta_{0j} + \beta_{1j}Yearsbeforedeath_{ij} + \beta_{[1]}Yearsbeforedeath_{ij}^2 + \boldsymbol{\beta_{[2]}I(TDP)_j} + \beta_{[3]}Ageatdeath_j + \beta_{[4]}I(APOEPos)_j + \beta_{[5]}I(Female)_j + \beta_{[6]}Education_j + \beta_{[7]}I(CAA)_j + \beta_{[8]}I(LB)_j + \beta_{[9]}I(NACCINF)_j + \boldsymbol{\beta_{[10]}Yearsbeforedeath_{ij} * I(TDP)_j} + e_{0ij}$$

Three-way interaction with time (Model B)

$$CogScore_{ij} = \beta_{0j} + \beta_{1j}Yearsbeforedeath_{ij} + \beta_{[1]}Yearsbeforedeath_{ij}^2 + \boldsymbol{\beta_{[2]}I(TDP)_j} + \boldsymbol{\beta_{[3]}I(ADNC)_j} + \beta_{[4]}Ageatdeath_j + \beta_{[5]}I(APOEPos)_j + \beta_{[6]}I(Female)_j + \beta_{[7]}Education_j + \beta_{[8]}I(CAA)_j + \beta_{[9]}I(LB)_j + \beta_{[10]}I(NACCINF)_j + \boldsymbol{\beta_{[11]}Yearsbeforedeath_{ij} * I(TDP)_j} + \boldsymbol{\beta_{[12]}Yearsbeforedeath_{ij} * I(ADNC)_j} + \boldsymbol{\beta_{[13]}I(TDP)_j * I(ADNC)_j} + \boldsymbol{\beta_{[14]}Yearsbeforedeath_{ij} * I(TDP)_j * I(ADNC)_j} + e_{0ij}$$

where,

$\beta_{0j} = \beta_0 + u_{0j}$
$\beta_{1j} = \beta_1 + u_{1j}$

$\beta_0 \sim N(0, 10)$

$\beta_1 \sim N(0, 10)$

Hierarchical Linear regression Model

- Identity link function relation between expected value of the response i.e. cognitive score and a linear function of the covariates (right hand side of the two-way and three-way models)
- $e_{0ij} \sim N(0, \sigma_{e0}^2)$
- $\sigma_{e0} \sim N(0, 2.5)$
- $\begin{bmatrix} u_{0j} \\ u_{1j} \end{bmatrix} \sim N(\begin{bmatrix} 0 \\ 0 \end{bmatrix}, \begin{bmatrix} \sigma_{u0}^2 & \sigma_{u0u1} \\ \sigma_{u0u1} & \sigma_{u1}^2 \end{bmatrix})$
- $LKJ\ prior\ for\ correlation\ matrix$

Hierarchical Beta regression Model

- Transform $CogScore_{ij}$ such that it lies between (0, 1)
- $CogScore_{ij} \sim beta(p, q)$; p, q>0
    - parameterisation : μ = p/(p + q) and ϕ = p + q such that $E(CogScore_{ij}) = \mu_{ij}$ and $Var(CogScore_{ij}) = \mu_{ij}(1 - \mu_{ij})/(1 + \phi)$
- Logit link function relation between expected value of the response i.e. cognitive score(μ) and a linear function of the covariates (right hand side of the two-way and three-way models)
- $\phi \sim cauchy(0, 5)$
- $u_{0j} \sim N(0, \sigma_{u0})$
- $u_{1j} \sim N(0, \sigma_{u1})$
- $\sigma_{u0} \sim N(0, 2.5)$
- $\sigma_{u1} \sim N(0, 2.5)$

i= repeated measures over time (Level 1)
j=individuals (Level 2)
$CogScore_{ij}$ = MMSE or CDR-SB score at each time point (visit)

$Yearsbeforedeath_{ij}$= Date of visit – Date of death (in years)

$I(TDP)_j$= Indicator variable equal to 1 when participant is TDP43+, 0 otherwise

$I(ADNC)_j$ = Indicator variable equal to 1 when participant is ADNC+, 0 otherwise

$Ageatdeath_j$ = Age of participant at death in years

$I(APOEPos)_j$ = Indicator variable equal to 1 when participant has atleast one APOE E4 allele, 0 otherwise

$I(Female)_j$ = Indicator variable equal to 1 when sex of participant is female, 0 otherwise

$Education_j$ = Years of education

$I(CAA)_j$ = Indicator variable equal to 1 when participant is CAA+, 0 otherwise

$I(LB)_j$ = Indicator variable equal to 1 when participant is Lewy Bodies+, 0 otherwise

I(NACCINF) = Indicator variable equal to 1 when participant is Infarct/Lacune+, 0 otherwise

β_{0j} = Random intercept for each participant

β_{1j} = Random slope for each participant

Mean posterior estimates and corresponding 95% credible intervals of **covariates mentioned in bold** above are listed in the tables below.

Supplementary Table S1: Hierarchical linear and beta regression estimates for comparing the effect of neuropathology groups on MMSE over time

Primary pathology	Model type	Parameter estimate for primary pathology (95% CI)[a]	Parameter estimate for (primary pathology * Time) (95% CI)[a]	Parameter estimate for ADNC (95% CI)[a]	Parameter estimate for (ADNC * Time) (95% CI)[a]	Parameter estimate for (primary pathology * ADNC) (95% CI)[a]	Parameter estimate for (primary pathology * ADNC * Time) (95% CI)[a]
TDP-43	2-way linear regression[b]	-3.82* (-6.17, -1.43)	-0.34[b] (-0.64, -0.04)				
	2-way beta regression[b]	-0.59* (-0.92, -0.29)	-0.03 (-0.07, 0.01)				
	3-way linear regression[c]	-9.07* (-13.01, -5.23)	-1.19* (-1.71, -0.69)	-11.49* (-14.17, -8.77)	-1.28* (-1.59, -0.96)	5.45* (0.83, 10.02)	0.86* (0.28, 1.45)
	3-way beta regression[c]	-1.55* (-2.11, -1.02)	-0.22* (-0.30, -0.13)	-1.78* (-2.13, -1.43)	-0.19* (-0.24, -0.14)	0.96* (0.30, 1.59)	0.19* (0.10, 0.28)
CAA	2-way linear regression[b]	-1.66 (-3.99, 0.61)	-0.12 (-0.42, 0.19)				
	2-way beta regression[b]	-0.20 (-0.49, 0.08)	-0.01 (-0.05, 0.02)				
	3-way linear regression[c]	-8.27* (-14.67,-1.62)	-0.89* (-1.67, -0.05)	-9.99* (-12.51,-7.61)	-1.07* (-1.40, -0.75)	6.57 (-0.28,13.29)	0.78 (-0.08, 1.65)
	3-way beta regression[c]	-1.41* (-2.15, -0.63)	-0.18* (-0.29, -0.06)	-1.63* (-1.94, -1.28)	-0.15* (-0.20, -0.11)	1.17* (0.34, 1.97)	0.16* (0.05, 0.28)
Lewy Body	2-way linear regression[b]	-2.31* (-4.58, -0.08)	-0.29* (-0.57, -0.01)				
	2-way beta regression[b]	-0.40* (-0.69,-0.10)	-0.07* (-0.11, -0.03)				
	3-way linear regression[c]	-1.26 (-5.49, 3.05)	-0.12[d] (-0.62, 0.40)	-9.32* (-12.14,-6.68)	-0.96* (-1.28, -0.62)	-0.98 (-5.61, 3.74)	-0.14 (-0.76, 0.41)
	3-way beta regression[c]	-0.11 (-0.62, 0.41)	0.02[d] (-0.05, 0.09)	-1.40* (-1.75, -1.05)	-0.10* (-0.15, -0.06)	-0.29 (-0.88, 0.33)	-0.09 (-0.17, 0.01)

Abbreviations: TDP, TDP-43; CAA, Cerebral Amyloid Angiopathy; LB, Lewy Bodies; ADNC, Alzheimer's disease neuropathological change

a Model was adjusted for main effect of time, time^2, age at death (years), binary APOE status, sex, education (years), the presence of infarcts and lacunes, and the presence of TDP-43, CAA, or Lewy bodies when not examined as a main effect.

b 2-way interaction between primary neuropathology and time before death (years) in ADNC+ participants

c 3-way interaction between binary neuropathology (TDP-43/ CAA/ Lewy Body), binary ADNC pathology and time before death (years)

d Scenario when there is disagreement between the direction of linear and beta regression's estimate

* 95% Credible intervals do not include the null value (zero)

Supplementary Table S2: Hierarchical linear and beta regression estimates for comparing the effect of neuropathology groups on CDR-SB over time

Primary pathology	Model type	Parameter estimate for primary pathology (95% CI)[a]	Parameter estimate for (primary pathology * Time) (95% CI)[a]	Parameter estimate for ADNC (95% CI)[a]	Parameter estimate for (ADNC * Time) (95% CI)[a]	Parameter estimate for (primary pathology * ADNC) (95% CI)[a]	Parameter estimate for (primary pathology * ADNC * Time) (95% CI)[a]
TDP-43	2-way linear regression[b]	3.22* (1.59, 4.76)	0.33* (0.13, 1.47)				
	2-way beta regression[b]	0.85* (0.49, 1.18)	0.07* (0.02, 0.11)				
	3-way linear regression[c]	5.49* (2.92, 8.04)	0.70* (0.35, 1.06)	6.83* (5.05, 8.59)	0.78* (0.56, 0.99)	-2.35 (-5.36, 0.66)	-0.38 (-0.78, 0.02)
	3-way beta regression[c]	1.82* (1.15, 2.49)	0.24* (0.14, 0.35)	2.07* (1.64, 2.51)	0.22* (0.16, 0.28)	-0.97* (-1.71, -0.20)	-0.18* (-0.30, -0.07)
CAA	2-way linear regression[b]	0.94 (-0.61, 2.54)	0.09 (-0.11, 0.29)				
	2-way beta regression[b]	0.13 (-0.22, 0.50)	0.01 (-0.28, 0.31)				
	3-way linear regression[c]	4.53* (0.27, 8.82)	0.50 (-0.02, 1.04)	6.41* (4.79, 8.18)	0.70* (0.48, 0.92)	-3.62 (-8.19, 0.77)	-0.42 (-0.98, 0.14)
	3-way beta regression[c]	1.48* (0.41, 2.44)	0.20* (0.05, 0.35)	1.93* (1.52, 2.39)	0.19* (0.13, 0.25)	-1.29* (-2.32, -0.19)	-0.18* (-0.34, -0.03)
Lewy Body	2-way linear regression[b]	1.68* (0.08, 3.35)	0.19 (-0.01, 0.39)				
	2-way beta regression[b]	0.42* (0.07, 0.76)	0.07* (0.02, 0.12)				
	3-way linear regression[c]	1.34 (-1.52, 3.98)	0.11[d] (-0.24, 0.45)	5.94* (4.09, 7.80)	0.63* (0.40, 0.86)	0.30 (-2.70, 3.32)	0.07 (-0.31, 0.48)
	3-way beta regression[c]	0.32 (-0.29, 0.93)	-0.03[d] (-0.13, 0.07)	1.74* (1.30, 2.17)	0.14* (0.07, 0.20)	0.12 (-0.54, 0.83)	0.10 (-0.01, 0.21)

Abbreviations: TDP, TDP-43; CAA, Cerebral Amyloid Angiopathy; LB, Lewy Bodies; ADNC, Alzheimer's disease neuropathological change

[a] Model was adjusted for main effect of time, time^2, age at death (years), binary APOE status, sex, education (years), the presence of infarcts and lacunes, and the presence of TDP-43, CAA, or Lewy bodies when not examined as a main effect.

[b] 2-way interaction between primary neuropathology and time before death (years) in ADNC+ participants

[c] 3-way interaction between binary neuropathology (TDP-43/ CAA/ Lewy Body), binary ADNC pathology and time before death (years)

[d] Scenario when there is disagreement between the direction of linear and beta regression's estimate

* 95% Credible intervals do not include the null value (zero)

Supplementary Figure S3: Posterior predictive checks comparing normal vs beta distribution assumptions for MMSE and CDR-SB scores

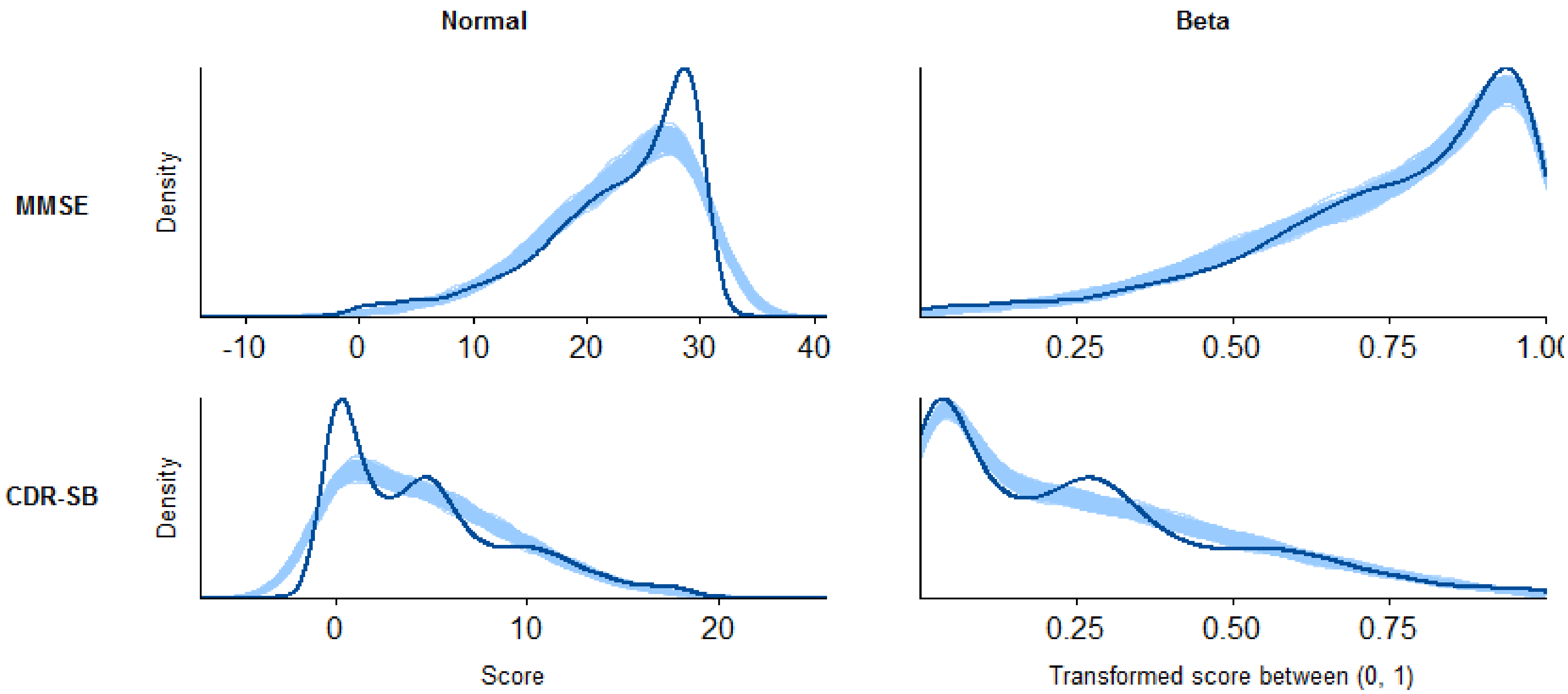

Abbreviations: MMSE, mini-mental state examination; CDR-SB, clinical dementia rating scale sum of boxes

Graphs represent a visual assessment of how well normal and beta distribution assumptions describe the observed distribution of MMSE and CDR-SB cognitive scores. Data were plotted using the bayesplot package in R. Dark line: Observed score density. Light lines: Draws from posterior predictive distribution.

Supplementary Figure S4: Sample selection flow chart

Sample selection step	Individuals	Visits
Complete data in NACC December 2018 data freeze	39412	136616
Individuals with an autopsy and data on ADNC, TDP-43, CAA and Lewy Bodies	924	4453
Exclusion based on presence of a familial dementia gene or rare neuropathology	701	3246
Logical checks of variables used	628	2472
Data on all variables (longitudinal scores, pathologies, and covariates)	574	2334

Abbreviations: TDP, TDP-43; CAA, Cerebral Amyloid Angiopathy; LB, Lewy Bodies; ADNC, Alzheimer's disease neuropathological change

This flow chart shows the data processing steps carried out to obtain our final analysis sample.
